# Correlation between the osteoporosis self-assessment tool for Asians index and QCT-derived bone parameters: a cross-sectional analysis of lumbar spine mineral density and content

**DOI:** 10.3389/fsurg.2026.1752041

**Published:** 2026-02-03

**Authors:** Cheng Li, Yang Lin, Wenyong Jiang, Ye Wang, Xuehui Chang, Fan Yang

**Affiliations:** Department of Orthopaedics, Guilin People’s Hospital, Guilin, Guangxi, China

**Keywords:** bone mineral content, bone mineral density, OSTA, QCT, spine, osteoporosis

## Abstract

**Objective:**

To correlate the Osteoporosis Self-assessment Tool for Asians (OSTA) index with lumbar QCT-measured bone mineral density (BMD) and bone mineral content (BMC), and assess its utility in evaluating lumbar spine BMD/BMC.

**Methods:**

From Jan 2023 to Jun 2024, 366 patients scheduled for lumbar spine surgery were enrolled. All underwent lumbar CT, QCT, and DXA. Spearman correlation analyzed OSTA vs. QCT-BMD and QCT-BMC. Patients were categorized by DXA T-scores into osteoporotic or non-osteoporotic groups. ROC curves were plotted to determine AUC, optimal cutoff, sensitivity, and specificity.

**Results:**

The OSTA index showed a moderate, statistically significant correlation with QCT-BMD and QCT-BMC (*P* < 0.01). Based on DXA diagnosis, QCT-BMC yielded AUCs of 0.712 (men) and 0.745 (women) for osteoporosis, indicating moderate diagnostic value. The optimal cutoffs were 663.86 mg for men and 521.65 mg for women, with sensitivities of 57.6% and 59.5%, respectively.

**Conclusion:**

As a screening tool, the OSTA index can help identify high-risk individuals undergoing spinal surgery who are likely to have low lumbar trabecular BMD and BMC as measured by QCT. Diagnostic thresholds for osteoporosis may be set at lumbar QCT-BMC <663.86 mg in men or <521.65 mg in women.

## 1 Introduction

In 2021, the United Nations General Assembly declared 2021 to 2030 as the “Decade of Healthy Ageing” highlighting musculoskeletal health as a key indicator of elderly health status. Osteoporosis, a representative disease of the musculoskeletal system in the elderly (1), has become increasingly prevalent due to increasing life expectancy and an ageing population. Over the past 40 years, the number of patients with osteoporosis in our country has reached 110 million. Concurrently, the incidence of osteoporotic fractures has increased annually, Posing significant societal and healthcare challenges. This necessitates heightened awareness among society, the public, and medical professionals (2). Effective tools for osteoporosis screening have thus become a focal point of current research (3). While dual-energy x-ray absorptiometry (DXA) remains the gold standard for diagnosing osteoporosis, quantitative computed tomography (QCT) offers a specialised bone mineral density (BMD) measurement technique capable of accurately assessing the volumetric density of cancellous and cortical bone. This provides a more comprehensive reflection of bone mass and the severity of osteoporosis (4).

The Osteoporosis Self-assessment Tool for Asians (OSTA) Index is a straightforward osteoporosis screening tool developed from a study that included data on age, body mass index, and bone mineral density from postmenopausal women in eight Asian countries and regions. It is increasingly being adopted in clinical practice. Initially, developed for postmenopausal women, subsequent studies have demonstrated its applicability to elderly men as well (5), The value of using the OSTA screening tool in other non-typical populations (aged 30 years or older) has also been investigated (6). Its utility in conditions complicated by osteoporosis has also been gradually recognised. Most previous research has focused on the relationship between OSTA and BMD measured by DXA. However, DXA evaluates the BMD of the entire vertebral body, including cortical and cancellous bone, which might result in false-positive increases in T-scores (7). The relationship between the OSTA index and true BMD and bone mineral content (BMC) remains unclear. Currently, limited studies have examined the correlation between the OSTA index and parameters measured by lumbar QCT, This study aims to analyse the applicability of the OSTA index in evaluating true BMD and BMC of the lumbar spine by investigating its correlation with parameters obtained from lumbar QCT.

## 2 Methods

### 2.1 Patient cohort

This study has been approved by the Ethics Committee of Guilin People's Hospital (Approval No.: 2024-241KY). Informed consent was waived due to the retrospective nature of the study. All methods were performed in accordance with relevant guidelines and regulations.

### 2.2 General information

This single-center retrospective study analyzed the demographic characteristics, clinical parameters, and treatment plans of 580 patients diagnosed with lumbar spine pathologies—including lumbar spinal stenosis, spondylolisthesis, and massive disc herniation with neurological deficits—who were scheduled for surgery at our institution between January 2023 and June 2024. Among them, 366 patients were scheduled for preoperative QCT or DXA based on the surgical team's judgment that a more precise assessment of bone quality was warranted, such as in cases planned for pedicle screw instrumentation or in patients with risk factors for osteoporosis. Ultimately, all 366 patients completed the imaging evaluations and met all inclusion criteria of this study, thereby constituting the final analytical cohort. Inclusion criteria: 1. Hospitalized for lumbar spine disease and concurrently underwent lumbar CT, QCT, and DXA (within 48 h); 2. Generally healthy lifestyle with no history of chronic heavy alcohol consumption or smoking, no severe limitations in daily activities, and a normal dietary structure. Exclusion criteria: 1. Presence of diseases that may affect bone metabolism, including but not limited to endocrine disorders (e.g., diabetes mellitus, hyperthyroidism, hypogonadism) or rheumatic autoimmune diseases; 2. Long-term use of medications that may influence bone metabolism, such as glucocorticoids, proton pump inhibitors, antiepileptic drugs, and aromatase inhibitors; 3. Structural spinal abnormalities that could interfere with bone density assessment, such as extensive enostosis (bone islands) or bone metastases; 4. Incomplete or poor-quality imaging data, lacking simultaneously valid lumbar CT, QCT, and DXA results.

### 2.3 DXA, CT, and QCT imaging and measurement

Patients undergoing DXA measurement (GE Lunar iDXA) should fast for ≥4 h to avoid interference from calcium supplements/contrast agents, remove metal objects, and wear cotton clothing without buttons/zippers. Standard positioning is adopted: supine position for the lumbar spine (L1-L4) with 15° internal rotation of the hips. A calibration phantom is scanned daily, with a coefficient of variation (CV) <1%. Vertebral endplates are automatically tracked, and osteophytes/degenerative areas are manually corrected. Reported parameters include BMD (g/cm^2^), T-score/Z-score (GE Lunar Normative Data for the Asian Population). For postmenopausal women and men aged 50 years and older, we utilized T-scores for diagnostic classification, adhering to the official criteria established by the WHO and the ISCD. The diagnostic categories were defined as follows: Normal: T-score ≥−1.0; Osteopenia: −2.5 < T-score <−1.0; Osteoporosis: T-score ≤−2.5. For premenopausal women and men under 50, assessment was performed using Z-scores. In accordance with ISCD guidelines, the diagnosis is not based on a specific cutoff value but rather integrated with the clinical context. In this study, we defined the categories as: “Below the Expected Range”: Z-score ≤−2.0; “Within the Expected Range”: Z-score >−2.0.

The patient underwent a lumbar spine dual-energy spectral CT scan (Philips Healthcare, Best, Netherlands) in the supine position using advanced technology. Scanning parameters were as follows: tube voltage 120 kV, tube current 125 mAs, pitch 1, slice thickness 1 mm, and table height 90 cm. Volumetric BMD measurements were performed, distinguishing between cortical/trabecular bone (ROIs avoided the vertebral venous plexus), with preference given to L1–L3 vertebrae (L4 was substituted in cases of fracture/artifact). The trabecular bone ROI was defined as an elliptical area 3 mm from the endplate and 2 mm from the inner edge of the cortical bone. The cortical bone ROI required manual verification of cortical continuity (thickness >1 mm) after automatic segmentation.

### 2.4 OSTA scores, QCT parameters, and T-scores

Height and body weight were measured by trained professionals. The OSTA score was calculated based on body weight and age using the formula: OSTA score = [body weight (kg)-age (years)] × 0.2. An OSTA value greater than −1 indicates low risk, a value between −4 and −1 (inclusive) suggests moderate risk, and a value below −4 signifies high risk. QCT parameters were obtained using dual-layer spectral CT with a fourth-generation QCT calibration phantom. The QCT-BMD and QCT-BMC values were determined as the average measurements of the lumbar 1 to lumbar 4 vertebral bodies.

### 2.5 Statistical analyses

The statistical analysis in this study was conducted using SPSS version 26 (IBM Corp., Armonk, NY, USA). Measurement data are shown as the means ± standard deviations. Normality of data distribution was assessed using the Kolmogorov–Smirnov test, with homogeneity of variance verified by Levene's test. Normally distributed datasets were analyzed with independent samples t-test, while non-normally distributed data were subjected to Mann–Whitney *U* tests. Due to non-normal data distribution, the Spearman correlation test was applied for OSTA index and QCT-BMD/BMC. Use the receiver operating characteristic(ROC) curve to assess utility and calculate corresponding sensitivity, specificity, and area under the ROC curve; AUC = 1 indicates complete diagnostic accuracy strength; 0.9 ≤ AUC < 1 indicates high diagnostic accuracy strength; 0.7 ≤ AUC < 0.9 indicates moderate diagnostic accuracy strength; 0.5 ≤ AUC < 0.7 indicates general diagnostic accuracy strength; AUC < 0.5 indicates no diagnostic accuracy strength. *P* < 0.05 was considered to indicate a statistically signifcant.

## 3 Results

**Figure 1:**
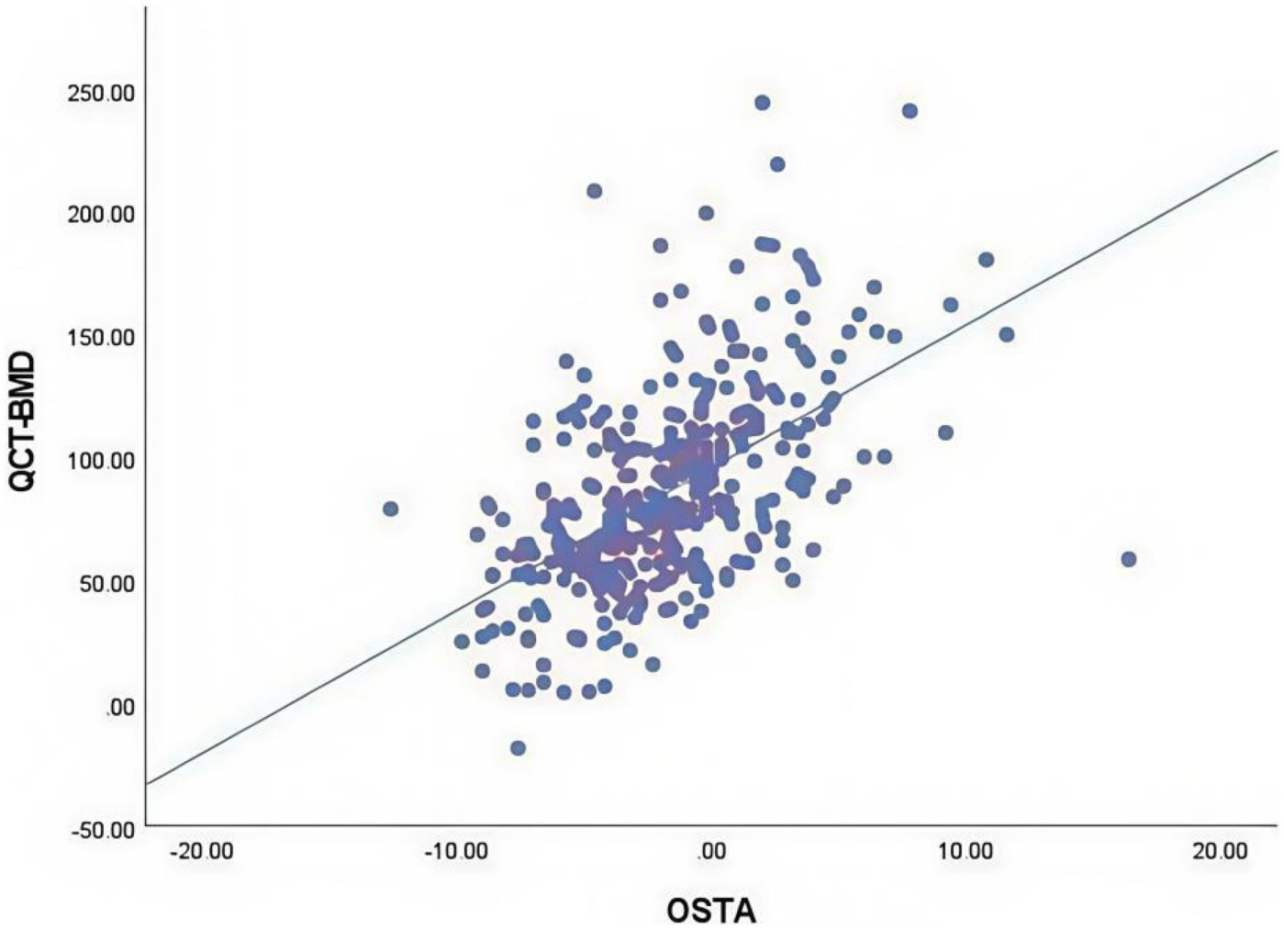
Moderate positive correlation of the QCT-BMD with the OSTA index (*R*^2^ = 0.305).

**Figure 2:**
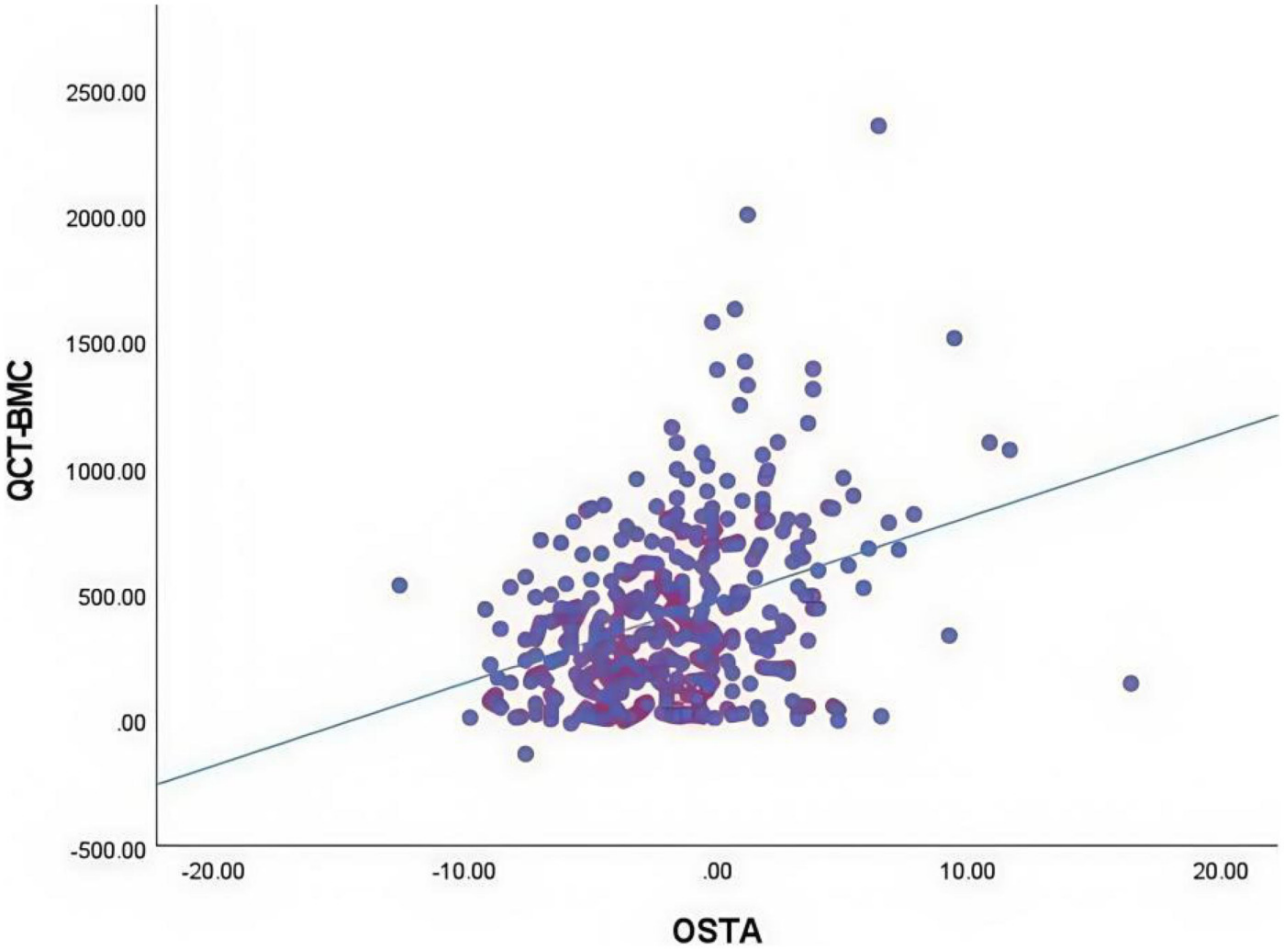
Modest positive correlation of the QCT-BMC with the OSTA index (*R*^2^ = 0.130).

**Figure 3:**
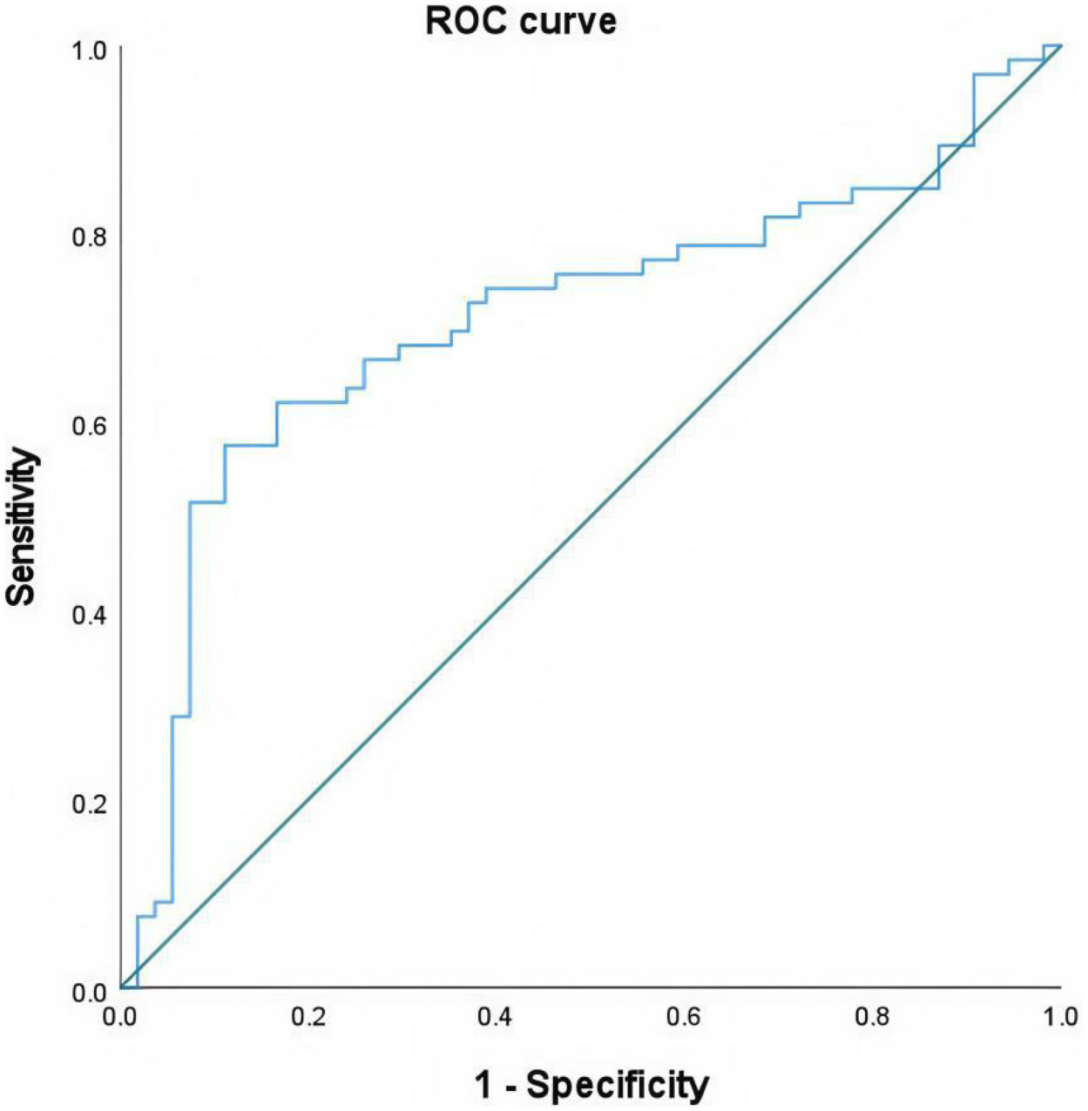
ROC curve of QCT-BMC in predicting osteoporosis in men.

**Figure 4:**
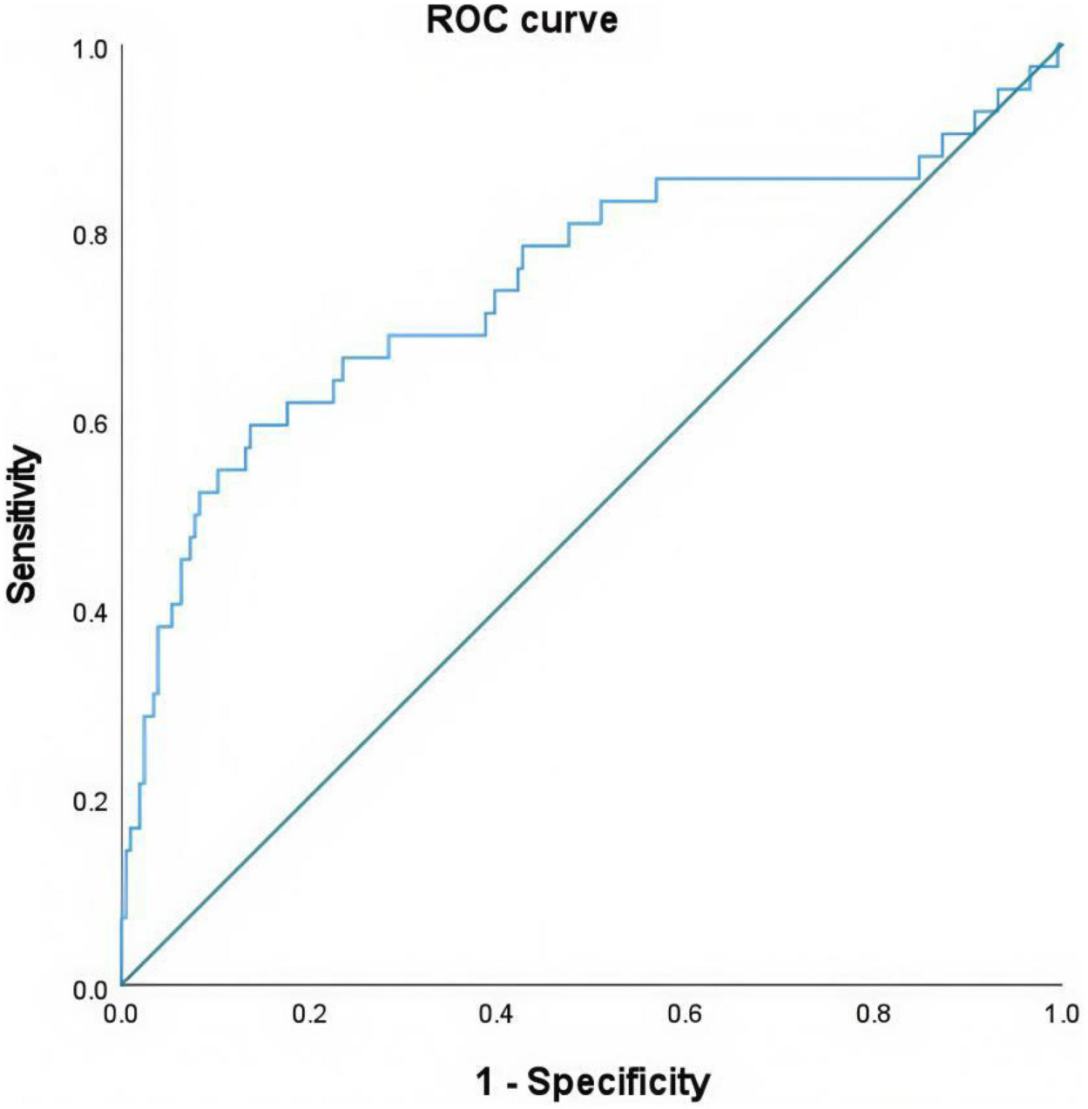
ROC curve of QCT-BMC in predicting osteoporosis in women.

A total of 366 patients were included in the final study, comprising 120 males with a mean age of 66.47 ± 12.14 years (range: 33–90) years and 246 females with a mean age of 68.41 ± 11.57 years (28–93) years (as shown in Table 1). The OSTA index demonstrated a moderate linear correlation with QCT-BMD (*r* = 0.552 *P* < 0.01) (Figure 1), and a modest linear correlation with QCT-BMC (*r* = 0.361 *P* < 0.01) (Figure 2) (as shown in Table 2). By using the osteoporosis status (defined as a DXA T-score <−2.5) as the classification variable and QCT-BMC as the test variable, a ROC curve was generated (Figures 3, 4). The results showed that QCT-BMC had an AUC of 0.712 (moderate diagnostic value) in men, with an optimal cutoff value of 663.86 and a sensitivity of 57.6%. In women, QCT-BMC achieved an AUC of 0.745 (moderate diagnostic value), with an optimal cutoff value of 521.65 and a sensitivity of 59.5% (as shown in Table 3).

**Table 1:** Baseline data of study patients.

Indicator	All (366)	Non-osteoporosis group (DXA T score ≥−2.5) (108)	Osteoporosis group (DXA T score <−2.5) (258)	*P*
Age	67.77 ± 11.78	59.80 ± 12.81	71.11 ± 9.53	<0.001
QCT-BMD	85.20 ± 40.47	131.72 ± 3.18	65.72 ± 1.50	<0.001
QCT-BMC	414.50 ± 350.98	647.63 ± 40.24	316.88 ± 16.44	<0.001
OSTA	−1.82 ± 0.20	0.81 ± 0.37	−2.92 ± 0.21	<0.001
BMI	23.63 ± 0.19	24.32 ± 0.37	23.34 ± 0.22	0.064
T-score	−3.36 ± 1.65	−1.37 ± 0.11	−4.20 ± 0.06	<0.001

**Table 2:** Correlation analysis between the OSTA index and QCT-BMD and QCT-BMC (*N* = 366).

Indicator	Method	OSTA	QCT-BMD	QCT-BMC
OSTA	Spearman correlation	1	.552^a^	.361^a^
	*P*		<0.001	<0.001
QCT-BMD	Spearman correlation	.552^a^	1	.552^a^
	*P*	.000		.000
QCT-BMC	Spearman correlation	.361^a^	.552^a^	1
	*P*	<0.001	<0.001	

a At the 0.01 level (two-tailed), the correlation is signifcant.

**Table 3:** With osteoporosis status (DXA T-score <−2.5) as the classification variable and QCT-BMC as the test variable, the ROC curve parameters were calculated.

Gender	AUC	Cut-off value	Sensitivity	Specificity	95% CI	*P* value
Man	0.712	663.86	0.576	0.889	0.617–0.807	<0.001
Woman	0.745	521.65	0.595	0.863	0.647–0.843	<0.001

## 4 Discussion

Osteoporosis has an insidious onset and often presents without significant symptoms in its early stages. Patients typically seek medical attention only after experiencing pain or fractures. Fragility fractures, the most common complication of osteoporosis, are highly detrimental and represent a leading cause of disability and death in elderly patients. Fragility fracture-related morbidity and mortality significantly affect global populations. In the United States, Canada, and Europe alone, approximately $48 billion has been spent on treating osteoporosis-related fractures (8). However, osteoporosis is preventable and treatable. Early screening and intervention can significantly reduce its associated harm (2). Therefore, early identification and screening of high-risk groups, along with efforts to reduce the incidence of osteoporosis and its complications, are essential for improving osteoporosis prevention, treatment, and management in China. The OSTA index, a simple and easy-to-calculate tool, has shown a moderate association with osteoporosis.

Currently, the availability of DXA in certain regions of China remains limited. Additionally, recent studies have highlighted certain limitations of DXA in osteoporosis screening and fracture risk assessment. For instance, British clinical guidelines advise against using DXA as a standalone tool for osteoporosis screening (9). The rapid advancements in QCT and CT offer valuable theoretical and practical support for their application in clinical practice, research, and health management related to osteoporosis (10). Investigating the OSTA index as a means to evaluate the true BMD and BMC of the lumbar spine is particularly crucial. In this study, lumbar QCT findings revealed a moderate linear correlation between the OSTA index and the true BMD and BMC of the lumbar spine. These results hold significant clinical relevance and guide for practice. As a simple, low-cost tool, the OSTA index has potential for widespread use in primary care, community-wide screening, and resource-limited areas. It is not a replacement for the gold-standard DXA but a complement to it. We aimed to validate the utility of OSTA as an effective preliminary screening tool to optimize the QCT-based risk assessment workflow. The goal is to better identify high-risk candidates for lumbar QCT scans, thereby improving the efficiency of clinical pathways and early diagnosis of osteoporosis.

### 4.1 Correlation between the OSTA index and BMD values measured using CT BMD

The OSTA index has demonstrated its utility in diagnosing osteoporosis among postmenopausal women in various Asian countries, including China, India, Japan, and Malaysia (11–13). However, these studies primarily rely on the gold standard DXA T-score of <−2.5. In contrast, this study addresses some limitations of DXA in assessing BMD and evaluates the true BMD of the lumbar spine using the OSTA index for the first time. Spearman correlation analysis reveals a moderate linear relationship between the OSTA index and lumbar QCT-BMD values in the studied population. Although the OSTA index demonstrated moderate correlation with QCT-BMD, its explained variance (*r*^2^ = 0.552) indicates approximately 45% of variability remains unaccounted for. Consequently, OSTA is more suitable as a preliminary screening tool in primary care settings, with positive cases requiring further confirmation through QCT or DXA.

### 4.2 Correlation between the OSTA index and BMC values measured using QCT-BMD

The OSTA index, derived from weight and age, incorporates two key factors influencing BMC. While weight significantly impacts BMC, it alone cannot provide an accurate assessment. Weight correction is necessary in osteoporosis diagnosis to minimise missed and incorrect diagnosis (14). El Hage et al. (15) analysed the BMC of adolescents with varying weights and found that obese boys exhibited lower apparent BMD compared to overweight and normal-weight boys (*P* < 0.05). In the elderly population, individuals with centripetal obesity and low muscle mass exhibit a markedly weak correlation between body weight and BMC (16). Age is another critical determinant of BMC. As cartilage ossification completes epiphyseal closure, osteogenic metabolism diminishes with age, resulting in a progressive decline in BMC. The OSTA index is derived from a combination of weight and age (17). In this study, Spearman correlation analysis revealed a significant correlation between the OSTA index and QCT-BMC (*P* < 0.01). This finding addresses limitations in previous studies wherein age, obesity, and sarcopenia affected the accuracy of weight-based BMC inferences. Furthermore, this study used DXA-derived T-scores to classify patients into osteoporosis and non-osteoporosis groups.

This study has several limitations. First, as a retrospective investigation involving only patients scheduled for lumbar surgery due to degenerative spinal conditions, the prevalence of osteoporosis may be underestimated, potentially introducing bias during data collection and analysis. Second, the study cohort was primarily recruited from northern Guangxi, which may not fully represent the demographic diversity across different regions of China. Future research should incorporate multi-center, multi-regional, and multi-ethnic samples to validate the reliability of these findings. Third, the OSTA was originally developed for postmenopausal women, whereas our study included male patients across various age groups. Future studies should employ stratified ROC analyses to address this limitation and yield more comprehensive results. Additionally, this study did not evaluate the long-term impact of OSTA-guided interventions on fracture incidence in the patient population. In recent years, the VBQ score derived from routine lumbar MRI has emerged as a promising non-invasive marker for assessing bone quality and osteoporosis risk in patients with degenerative diseases (18). Direct comparisons of OSTA with tools such as VBQ and QCT in terms of diagnostic performance, cost-effectiveness, and convenience across different clinical scenarios would help refine personalized osteoporosis risk stratification and management pathways for patients with lumbar disorders. Further interventional studies are warranted to address this knowledge gap.

In conclusion, the OSTA index, calculated using weight and age, demonstrates a moderate linear correlation with BMD and BMC values measured by QCT. The OSTA index may serve as a preliminary risk stratification or screening tool for osteoporosis, rather than a substitute for imaging-based assessments such as DXA or QCT, by systematically validating the correlation between OSTA and QCT-derived BMC in a large-scale cohort, our work offers compelling evidence supporting the integration of OSTA into QCT-based osteoporosis assessment protocols. DXA serves as a practical clinical reference for trabecular bone quality rather than a gold standard, as it measures the bone mineral density of the entire vertebral body (including both cortical and trabecular bone). Owing to its measurement principles, QCT may offer greater sensitivity in detecting age- or treatment-related changes in bone density. In patients scheduled for spinal surgery, a lower OSTA index is associated with reduced lumbar BMC and BMD, whereas a higher index indicates greater values. Notably, Osteoporosis may be indicated when lumbar QCT-BMC values fall below 663.86 mg in men or 521.65 mg in women. However, since the study focused specifically on clinical patients requiring lumbar spine surgery, its sensitivity may not generalize to all clinical scenarios. Therefore, practical application should incorporate clinical judgment.

## Data Availability

The raw data supporting the conclusions of this article will be made available by the authors, without undue reservation.
